# Combining Detailed Fetal Anatomy Scanning in the NT Window Versus Early Second Trimester Scanning at 14–16 Weeks: A Non‐Inferiority Study

**DOI:** 10.1002/jum.70036

**Published:** 2025-08-26

**Authors:** Tomer Shwartz, Sarah M. Cohen, Michal Lipschuetz, Hila Hochler, Yaron Zalel, Dan V. Valsky, Simcha Yagel

**Affiliations:** ^1^ Department of Obstetrics and Gynecology Hadassah University Medical Center Jerusalem Israel; ^2^ Faculty of Medicine of the Hebrew University of Jerusalem Jerusalem Israel; ^3^ Henrietta Szold Hadassah Hebrew University School of Nursing in the Faculty of Medicine Jerusalem Israel; ^4^ Department of Obstetrics and Gynecology Laniado Medical Center Netanya Israel; ^5^ Adelson School of Medicine Ariel University Ariel Israel; ^6^ Obstetrics and Gynecology, Expert Medical Center Tel‐Aviv Israel

## Abstract

**Objectives:**

First‐trimester ultrasound has evolved to incorporate a detailed fetal anatomy scan (FAS) with nuchal translucency (NT) screening. Many institutions use a 2‐visit protocol: NT followed by detailed FAS at 14–16 weeks. We aimed to evaluate whether integrating detailed FAS into the NT window (12 + 5 to 13 + 6 weeks) is non‐inferior in diagnostic yield to the 2‐visit protocol.

**Methods:**

We enrolled 755 mixed high‐ and low‐risk pregnant women with singleton gestations into either integrated NT + FAS group (n = 243, 12 + 5 to 13 + 6 weeks) or 2‐visit group (n = 512, 14–16 weeks). All underwent follow‐up at 20–24 weeks. Scans followed ISUOG guidelines; the primary outcome was detection of fetal anomalies during detailed early scans. Non‐inferiority was defined as a margin of ≤6% difference in detection rates.

**Results:**

Study groups were similar except in parity (mean 2.6 [0–9] vs. 3.1 [0–10], *P* = .0081 in FAS + NT and 2‐visit group, respectively). Anomalies were identified in 16 fetuses (7.5%) in the NT + FAS group and 16 (3%) in the 2‐visit group (*P* = .033). Additional anomalies were detected at mid‐trimester in 2/227 (0.9%) NT + FAS and 4/496 (0.8%) 2‐visit group fetuses (*P* = 1.0). The integrated approach met non‐inferiority criteria.

**Conclusion:**

Detailed FAS during the final NT window week is non‐inferior to the 2‐visit approach. This integrated protocol offers earlier reassurance without compromising diagnostic accuracy.

**Abbreviations**
AV‐canal: atrioventricular canal defect
BMI: body mass index
BPC: Blake's pouch cyst
CNS: central nervous system
FAS: fetal anatomy scanning
GA: gestational age
Mmc: myelomeningocele
NT: nuchal translucency
PA: pulmonary arteries
SVT: supraventricular tachycardia
TA: truncus arteriosus
TGA: transposition of the great arteries
TOP: termination of pregnancy
TV: tricuspid valve
TVS: transvaginal scan(ning)
VSD: ventricular septal defect

Over the last two decades, fetal first trimester screening has evolved beyond nuchal translucency for genetic and structural anomaly screening to include fetal anatomic diagnostic scanning. In the most recent ISUOG guideline for first trimester screening at 11–14 weeks gestational age,^1^ two approaches to first trimester anatomy scanning are described. The basic anatomy scan, in addition to crown–rump length, nuchal translucency (NT), and biparietal diameter, includes examination of the head and brain, the neck, heart, abdomen, spine, extremities, and the placenta.^1^ The detailed anatomy scan includes the basic components while adding the face, thorax, amniotic fluid and membranes, and specified target anatomy in each organ system.^1^

First trimester anatomy scanning has been investigated^2^, ^3^ for its diagnostic yield in anomaly detection. In their systematic review, Karim et al showed a wide range in detection rates of fetal anomalies in the first‐trimester scan, ranging from 32% in low‐risk groups to more than 60% in high‐risk groups,^2^ and that a standardized protocol increased scan yield.^2^ The same group performed a systematic review of first‐trimester diagnosis of fetal heart defects and found that while over half were diagnosed in first‐trimester scanning, there was variation in diagnosis rates among different types of lesions. Here too, the investigators found that scanning protocols significantly affected the screening performance.^4^ Syngelaki et al^3^ in their large study also found wide variation in the diagnosis rates of anomalies, showing that some defects were not amenable to diagnosis during this scan window.^3^ They also pointed out the importance of a standardized scanning protocol to optimize performance.^3^

In the context of screen‐positive NT (NT > 99th centile), Zalel et al^5^ demonstrated the feasibility of offering early detailed anatomy scanning, prior to genetic evaluation. Scans were performed at a mean GA of 12.4 weeks; major sonographic findings were present in almost 70% of fetuses.

In our 2015 study,^6^ we demonstrated that during the early NT window (before 12 weeks of gestation or a CRL of 62 mm), much of the target anatomy assessed in a detailed anatomy scan is not yet feasibly accessible for imaging.^6^

In our institutions and many others, the late first trimester/early second trimester targeted organ scan has been performed at 14–16 weeks since the implementation of transvaginal scanning.^7^ The scan protocol meets or exceeds the requirements for detailed first trimester scan as recommended by ISUOG.^1^ When this approach was first applied, transducer and machine capabilities were insufficient to meet the demands of first‐trimester anatomy scanning beyond the basics. With the advancements in ultrasound technology, including higher‐resolution transducers and improved imaging systems, it may be feasible to combine the NT screening visit and early targeted organ scan into general practice, with the caveat that the scan be performed in the last week of the NT window.^6^

The two‐visit protocol currently in use in our health system (i.e., NT screening at 11–13 weeks followed by early targeted organ scan at 14–16 weeks of gestation) can be logistically burdensome for patients and inefficient for caregivers, potentially creating barriers to accessing or providing care. Caregivers may be hesitant to integrate the NT and fetal anatomy scans out of an abundance of caution regarding screening performance. Earlier confirmation of normal fetal anatomy at the time of the NT scan provides reassurance to patients and, in cases where abnormalities are detected, facilitates timely genetic counseling and testing when chorionic villus sampling remains possible, enabling earlier decision‐making regarding pregnancy continuation.

We sought to perform a non‐inferiority study to show that moving detailed anatomy scanning from the 14–16 weeks window to the last week of the NT screening window would not detract from the diagnostic yield of the anatomy scan. By demonstrating non‐inferiority in anomaly detection, we aimed to validate a one‐step approach that is both patient‐centered and clinically effective.

## Methods

### Study Design

This was a pilot, proof‐of‐concept, non‐inferiority study comparing two approaches to first‐trimester screening and detailed fetal anomaly scanning (FAS): the study group received NT screening integrated with early detailed fetal anatomical scanning during the latter part of the NT window (12 weeks + 5 days to 13 weeks + 6 days) while the comparison group underwent nuchal translucency (NT) screening during the NT window, followed by detailed FAS at 14–16 weeks of gestation. All participants also underwent a routine detailed mid‐trimester FAS between 20 and 24 weeks. The study employed a parallel group design.

### Study Population

Eligible participants were pregnant individuals aged 18–49 presenting for NT screening with singleton pregnancies and no prior diagnosis of fetal anomalies. Exclusion criteria included multiple gestations and known chromosomal abnormalities. As a referral center, our patient population is comprised of a mix of low‐ and high‐risk patients.

### Recruitment and Allocation

Participants were recruited through social media forums and clinical sites and were allocated to either the integrated NT + FAS approach or the standard two‐visit protocol based on their preference. A total of 755 participants were enrolled: 243 in the study group underwent NT + FAS screening between 12 + 5 and 13 + 6 weeks of gestation, and 512 in the comparison group consisted of those preferring to delay FAS to 14–16 weeks for scheduling or personal reasons, including a preference to avoid transvaginal scanning or presenting for NT screening earlier in the NT window. Patients with suspected anomalies in the NT scan were excluded from the study.

### Scan Procedure

All scans adhered to the guidelines published by ISUOG for the performance of the 11‐ to 14‐week ultrasound scan. The first‐trimester scan included confirming fetal viability, accurate pregnancy dating via crown‐rump length (CRL) measurement, confirming singleton pregnancy, and NT thickness, Doppler assessment of uterine arteries and maternal biochemical screening, as well as FAS targets as described in the guideline for detailed anatomy scan^1^ (online supplemental Table 1) and investigation of the intracranial translucency^8^, ^9^ and fetal heart screening as described in the ISUOG guidelines (updated) for fetal heart screening examination.^10^ Among comparison group patients, NT thickness, Doppler assessment of uterine arteries, and maternal biochemical screening studies were performed prior to their presentation for their 14–16 weeks scans, which included the same target elements as the study group.

The mid‐trimester detailed fetal anatomical scans were performed in accordance with the updated ISUOG and other societies' guidelines,^11^, ^12^, ^13^ and included assessment of fetal viability, biometry, and detailed anatomical evaluation, including the head and brain, face, neck, chest and heart, abdomen, skeleton, genitalia, placenta, umbilical cord, and cervix, as well as heart examination following the ISUOG guidelines for fetal heart screening^5^ and other recognized echocardiography guidelines.^14^ In addition to transabdominal ultrasonography, transvaginal scans were performed as needed to optimize imaging of the cervix or other structures.

### Equipment

All scans were performed using a GE E22 ultrasound machine with advanced transabdominal and transvaginal probes (RIC6‐12‐D, RIC5‐9‐D, C2‐9‐D, RM7C). Vaginal scanning was utilized when necessary to optimize image quality, particularly for early FAS.

### Outcomes

The primary outcome was the diagnostic yield of fetal anomalies identified at early detailed FAS compared with the standard two‐visit approach. Secondary outcomes included patient acceptability, defined as the proportion of patients agreeing to the integrated approach and the need for transvaginal scanning.

### Sample Size and Statistical Analysis

The study enrolled 243 participants in the study group and 512 in the comparison group. Power analysis for the full‐scale non‐inferiority trial was performed to determine the adequacy of sample size. Descriptive statistics were used to summarize diagnostic yield and patient acceptability. Non‐inferiority was assessed using a predefined margin of 6% difference in success rates.^15^

### Ethical Approval

The study protocol was approved by the Institutional Review Board (Helsinki Committee of the Hadassah Medical Organization on 13 July 2023, approval no. 0232‐23‐HMO). All participants provided informed consent for themselves and their fetuses, prior to inclusion in the study. Patient confidentiality was maintained throughout, with anonymized data used for analysis.

## Results

A total of 755 participants were enrolled in the study: 243 in the study group that underwent NT integrated with early detailed FAS between 12 + 5 and 13 + 6 weeks' GA, and 512 in the comparison group, who received the standard 2‐visit protocol, with detailed FAS at 14–16 weeks.

Table 1 shows the background parameters of the study participants. No statistically significant differences were found between the groups except for parity, which was slightly higher in the comparison group. Notably, maternal BMI, which may impact scan quality, did not differ between study and comparison groups (*P* = 1.0).

**Table 1:** Obstetric Background Parameters of the Study and Comparison Groups

Parameter	FAS in NT Window N = 243	FAS 14–16 Weeks N = 512	*P*‐Value
Gestational age at scan, weeks [median, (range)]	13^+1^ (12^+5^–13^+4^)	15^+4^ (14^+6^–16^+4^)	.3
Age	29.4 (19–43)	31.7 (20–44)	.7
Parity [median, (range)]	2.6 (0–9)	3.1 (0–10)	.0081
BMI	24.3 (18–36)	24.8 (18–36)	1.0
Scan approach			
Abdominal only	14 (5.8%)	444 (86.7%)	<.001
Abdominal + Vaginal	229 (94.2%)	68 (13.2%)	
Duration of scan [min, mean, (range)]	22 (17–51)	24 (16–55)	1.0
GA at mid‐trimester scan	22^+1^ (21^+3^–23^+1^)	22^+1^ (21^+2^–23^+4^)	1.0

### Primary Outcome

**Figure 1:**
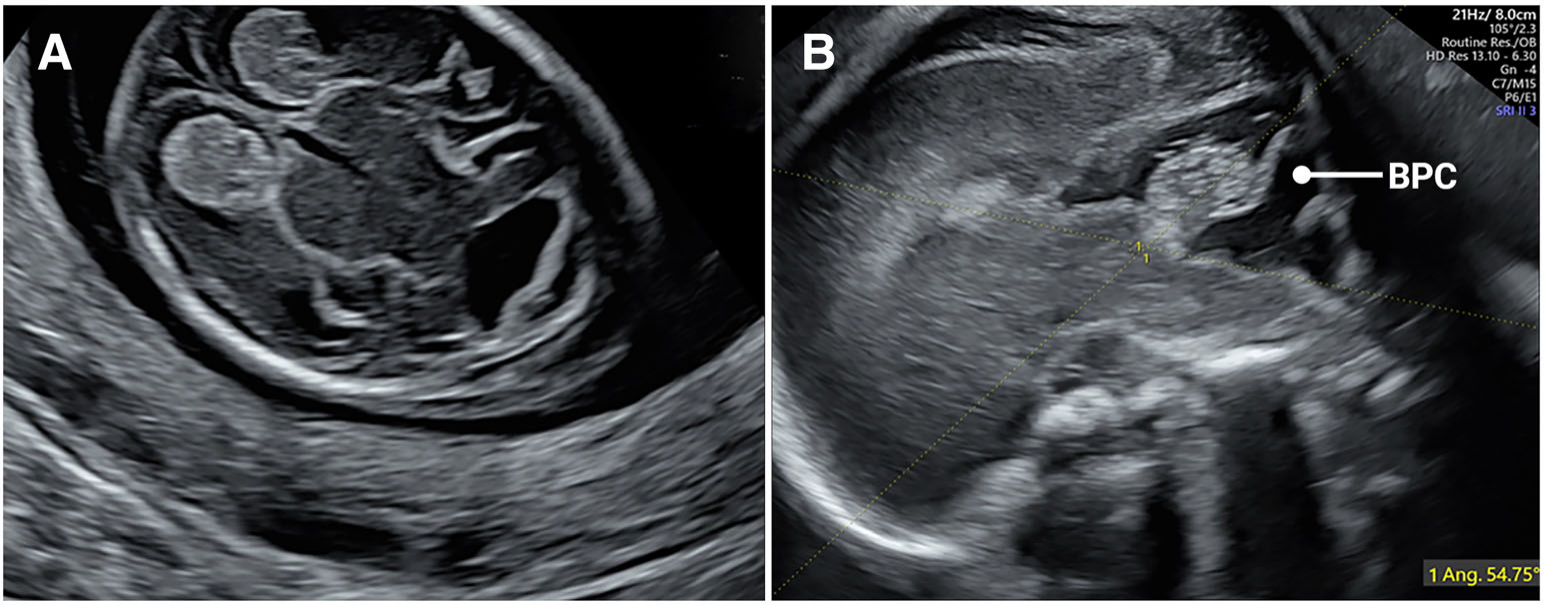
Early diagnosed Blake's pouch cyst (BPC). **A**, Enlarged 4th ventricle at 13^+2^ weeks raised suspicion for BPC. **B**, Midtrimester scan at 23 weeks confirmed the 1st trimester diagnosis (BPC, Blake's pouch cyst).

**Figure 2:**
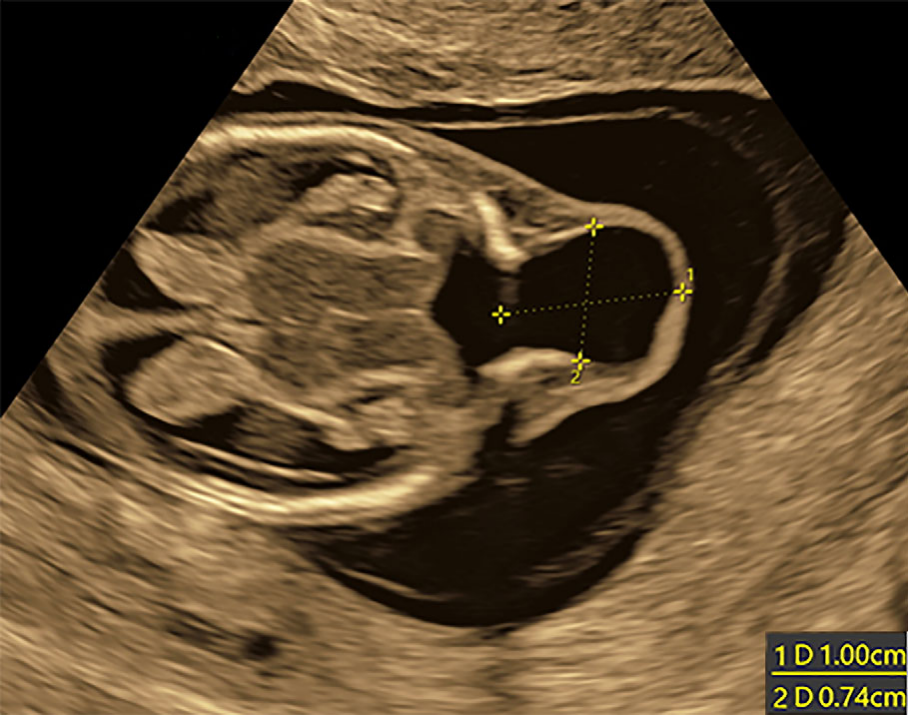
Encephalocele diagnosed during the early detailed scan at 13^+3^. Calipers show the extent of the lesion.

**Figure 3:**
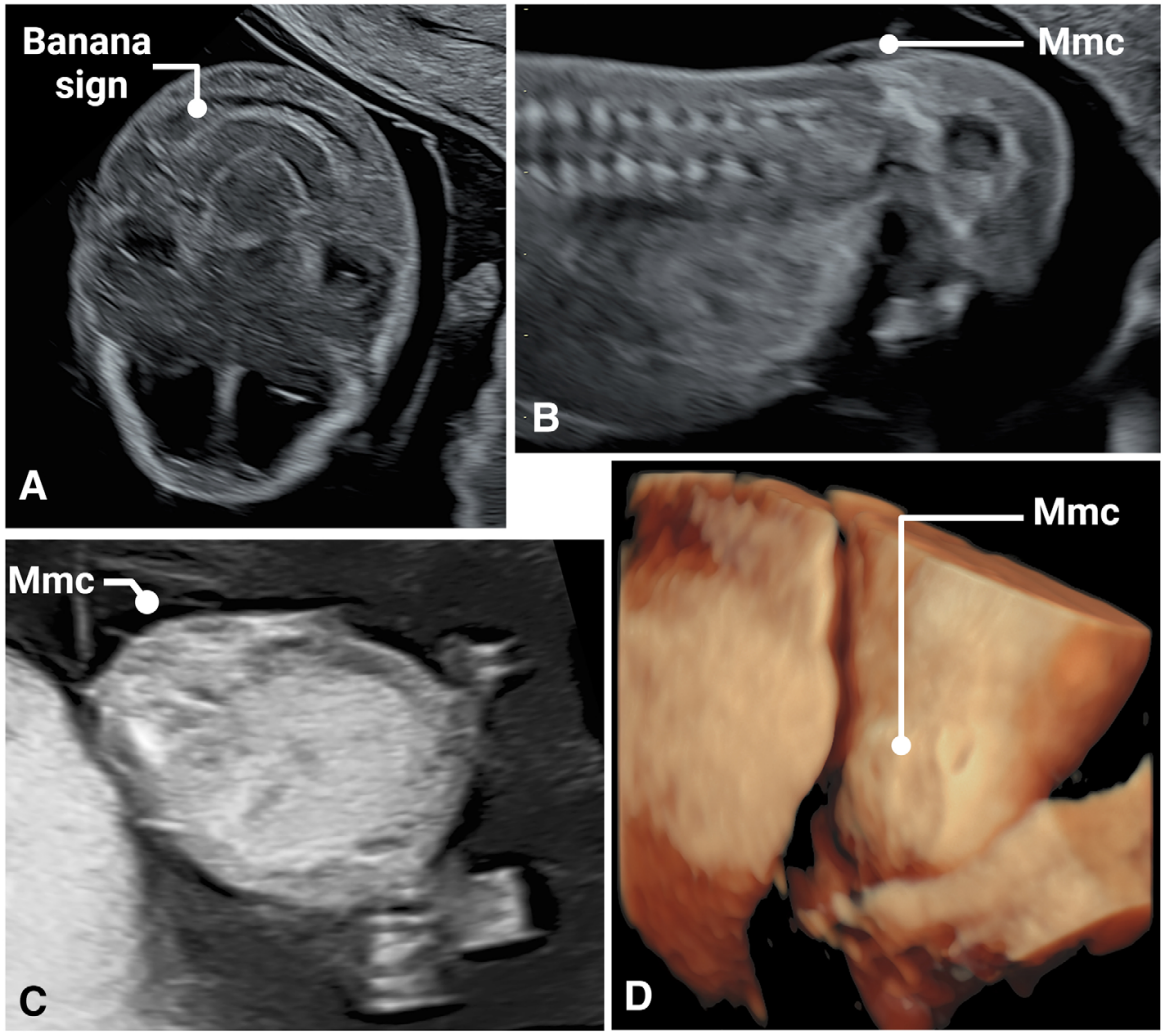
Myelomeningocele was suspected when the characteristic banana sign was diagnosed at 13^+3^ weeks (**A**). The membrane was visualized protruding from the lumbar region in longitudinal and transverse scans (**B** and **C**). Myelomeningocele imaged with 3D rendering (**D**) (Mmc, myelomeningocele).

**Figure 4:**
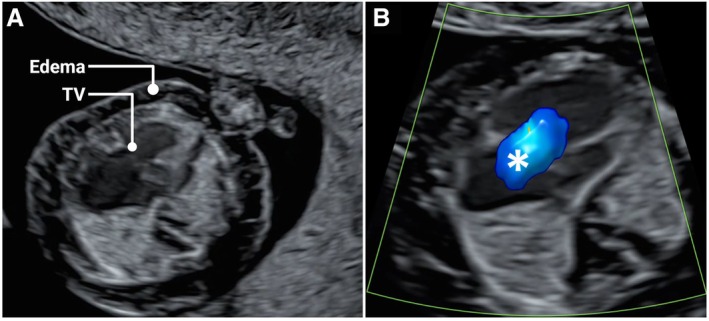
Ebstein anomaly was diagnosed at 12^+5^ weeks in this fetus with marked edema and tricuspid valve insufficiency (**A**). Color Doppler showed tricuspid regurgitation during systole (asterisk) (**B**) (TV, tricuspid valve).

**Figure 5:**
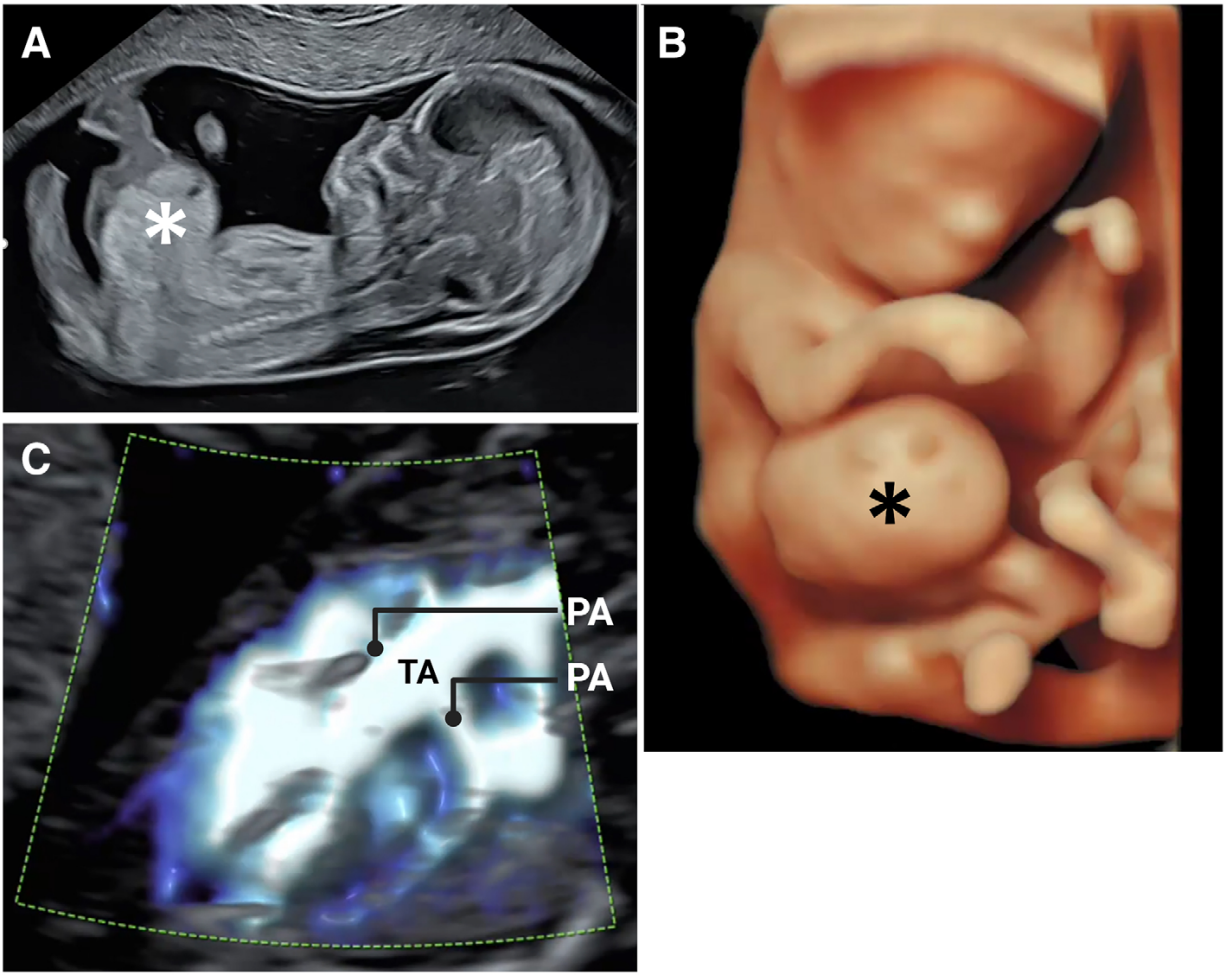
A complex case of truncus arteriosus with omphalocele, diagnosed at 12^+6^ weeks. The omphalocele (*) is imaged in the longitudinal plane (**A**) and with 3D rendering (**B**). The truncus arteriosus (Type II) is demonstrated with the Slow*flow*HD ultrasound modality (**C**) (PA, pulmonary arteries; TA, truncus arteriosus).

As a referral center, our population is mixed low‐ and high‐risk for fetal anomalies. As such, we detected a higher‐than‐expected rate of anomalies during the fetal anatomy scans in both study groups. Table 2 lists the identified anomalies and their outcomes up to the mid‐trimester scan. A total of 34 fetuses were diagnosed with structural anomalies during the initial scan in both the study and comparison groups: 18 (7.4%) in the NT + FAS group, and 16 (3%) in the comparison group (*P* = 0.13, Fisher's exact test for rate of anomalies). Figures show selected cases of early‐diagnosed anomalies: Blake's pouch cyst (Figure 1, A and B), Encephalocele (Figure 2, online supplemental Video 2), Myelomeningocele (Figure 3, A–D, online supplemental Video 3b,c), Ebstein anomaly (Figure 4, A and B, online supplemental Video 4a,b), and Truncus with omphalocele (Figure 5, A–C, online supplemental Video 5a–c).

**Table 2:** Anomalies Diagnosed in the Study and Comparison Groups

	FAS in NT Window	Outcome	FAS 14–16 Weeks	Outcome
Brain/CNS	Acrania	TOP	Cephalopagus	TOP
	Anencephaly	TOP	Holoprosencephaly	TOP
	Blake's pouch cyst (Figure 1, A and B)	Ongoing	Hydrocephaly	TOP
	Encephalocele (Figure 2, online supplemental Video 2)	TOP	Hydrocephaly w/right aortic arch	TOP
	Myelomeningocele (Figure 3, A–D, online supplemental Video 3b,c)	TOP	Meckel‐Gruber syndrome	TOP
Face	Bilateral cleft lip	Ongoing	Bilateral cataract	Ongoing
Heart	AV‐canal w/NT 9 mm	Trisomy 21, TOP	Aberrant right subclavian artery	Ongoing
	AV‐canal	Ongoing	Agenesis of IVC	Ongoing
	Ebstein anomaly	Ongoing	Heterotaxy (right isomerism w/AV‐canal)	Trisomy 21, TOP
	Ebstein anomaly (Figure 4, A and B, online supplemental Video 4a,b)	TOP	Hypoplastic left heart	TOP
	Hypoplastic left heart	TOP	Supraventricular tachycardia (SVT)^a^	TOP
	Hypoplastic left heart w/cystic hygroma	Trisomy 18, TOP	Tetralogy of Fallot w/hydrocephaly and cleft lip	TOP
	Mitral atresia w/VSD	Ongoing	TGA w/pulmonary stenosis	Ongoing
	Tetralogy of Fallot	Ongoing		
	Tetralogy of Fallot	Ongoing		
	Tetralogy of Fallot	Ongoing		
	Truncus with omphalocele (Figure 5, A–C, online supplemental Video 5a–c)	TOP		
Thorax			Chylothorax	Ongoing
			Left diaphragmatic hernia	Ongoing
			Left diaphragmatic hernia	Ongoing
Abdomen	Gastroschisis	Ongoing		

^a^ SVT was refractory to transplacental pharmacological treatment. AV‐canal, atrioventricular canal defect; TGA, transposition of the great arteries; TOP, termination of pregnancy; VSD, ventricular septal defect.

A total of six fetuses were diagnosed with structural anomalies during the mid‐trimester scans (21–23 weeks) that were not seen during the earlier scans (Table 3). In the FAS + NT group, two cases were diagnosed (2/225), one agenesis of the corpus callosum and one rhabdomyoma. In the comparison group, four fetuses were diagnosed with previously undetected anomalies (4/496): one each of partial agenesis of the corpus callosum, aortic stenosis, chylothorax, and bilateral clubfoot (*P* = 1.0, Fisher's exact test for rate of newly diagnosed anomalies). The results confirmed the diagnostic yield of the early integrated NT + FAS approach as non‐inferior to the standard two‐visit protocol.

**Table 3:** Anomalies Detected at the Mid‐Trimester Scans at 21–24 Weeks, in the Study and Comparison Groups

	FAS in NT Window	FAS 14–16 Weeks
Brain/CNS	Agenesis of corpus callosum	Partial agenesis of corpus callosum
Heart	Rhabdomyoma	Aortic stenosis
Abdomen		Chylothorax
Limbs		Bilateral clubfoot

### Secondary Outcomes

The early integrated NT + FAS approach demonstrated comparable patient acceptability to the standard protocol. The proportion of scans requiring transvaginal imaging differed between the groups (*P* < 0.001), reflecting the difference in fetal dimensions. We encountered comparable satisfaction with the integrated approach and standard protocol, of 96%, and very rare non‐compliance to undergo TVS when indicated (4 patients in each group, 0.5%).

## Discussion

This study demonstrates that integrating detailed fetal anatomical scanning into the final days of the NT screening window (12 + 5 to 13 + 6 weeks of gestation) does not compromise the diagnostic yield of detecting fetal anomalies compared with a two‐visit protocol that delays detailed FAS to 14–16 weeks. Furthermore, the integrated approach proved acceptable to patients, both in terms of logistics and the necessity of transvaginal scanning.

The findings align with and expand on ours^6^ and others'^2^, ^3^, ^4^, ^5^ previous research evaluating the feasibility and diagnostic accuracy of early detailed FAS. We highlighted^6^ the potential of detailed transabdominal fetal anatomical scanning in the late first trimester, noting its diagnostic accuracy was not inferior to scans performed in the early second trimester when high‐resolution imaging technology and expertise are utilized, including transvaginal scanning as indicated. This supports our observation that advancements in ultrasound technology allow for effective visualization of fetal structures during the NT window.

Syngelaki et al^3^ demonstrated the feasibility of detecting non‐chromosomal abnormalities as early as 11–13 weeks; however, they detected only 14% of subtle structural anomalies, up to 90% in different organ systems, and reaching 100% of some severe anomalies.^3^ These results underscore the value of first‐trimester FAS not only for early reassurance but also raise questions regarding scan effectiveness for some lesions. Our results, showing a comparatively higher diagnostic yield, emphasize the importance of waiting for the latter part of the NT window to examine the more‐developed fetus,^6^ as well as the advantage of the transvaginal scanning approach to visualize subtle findings. In screen‐positive NT fetuses, when the early detailed anatomy scan is performed with a high degree of suspicion, Zalel et al^5^ also demonstrated the high yield of the examination. Their findings showed that severe structural abnormalities were predictive of aneuploidy, with an adjusted odds ratio of 8.15.^5^

Karim et al^2^ emphasized the importance of systematic first‐trimester screening protocols and their ability to detect structural anomalies effectively, particularly when operators follow standardized protocols, such as those established by ISUOG, as was the case in our study. In low‐risk and unselected populations, the pooled sensitivity of first trimester ultrasound was about 32.4%. Use of a systematic protocol increased sensitivity across all subgroups of anomalies and risk levels. We applied the same standardized protocol to both scanning groups and showed that the NT + FAS scan window had similar rates of anomalies and diagnostic yield as the later FAS window.

The ability to diagnose fetal heart anomalies early is particularly significant. Karim et al^4^ conducted a systematic review and meta‐analysis highlighting the high diagnostic yield of first‐trimester ultrasound in identifying cardiac abnormalities. They showed a pooled sensitivity of 55.8% and a positive predictive value of 94.85% to detect fetal cardiac anomalies in the first trimester.^4^ We showed that in our small study group, the diagnosis rate for cardiac defects was not negatively impacted among the earlier scan group.

Patient acceptability and access are other considerations. Our study addressed a key patient‐centered concern: reducing the logistical challenges of multiple appointments while maintaining high diagnostic standards. The integrated NT + FAS approach offers earlier reassurance to patients and ensures timely genetic investigations or decisions, such as pregnancy interruption where legally permitted. Non‐compliance with the performance of indicated TVS was encountered only rarely.

In summary, this study confirms that moving FAS into the NT window maintains diagnostic accuracy and aligns with patient preferences. These findings are consistent with prior literature, demonstrating that first‐trimester ultrasound, when performed in the final week of the NT window, with advanced imaging systems and adhering to ISUOG protocols, can effectively meet the dual goals of early diagnosis and patient‐centered care. Future research should explore broader implementation strategies and long‐term outcomes to further validate and optimize this approach.

## Supporting information


**Supplemental Table 1.** A systematic approach to detailed assessment of the fetal anatomy at 11^+0^ to14^+0^ weeks should include the following.

**Supplemental Data 1.** Supplement to Figure 2, Encephalocele: online supplemental Video 2: Encephalocele diagnosed during the early detailed scan at 13 + 3.

Supplement to Figure 3, Myelomeningocele: online supplemental Videos 3b and 3c: The membrane visualized protruding from the lumbar region in longitudinal and transverse scans.

Supplement to Figure 4, Ebstein anomaly: online supplemental Video 4a: Ebstein anomaly was diagnosed at 12 + 5 weeks in this fetus with marked edema and tricuspid valve insufficiency. 4b: Color Doppler showed tricuspid regurgitation during systole.

Supplement to Figure 5, Truncus with omphalocele, online supplemental Video 5a: A complex case of truncus arteriosus with omphalocele, diagnosed at 12 + 6 weeks. The omphalocele imaged in longitudinal plane. 5b: The omphalocele imaged with 3D rendering. 5c: The truncus arteriosus (Type II) is demonstrated with the SlowflowHD ultrasound modality.

## Data Availability

The data that support the findings of this study are available on request from the corresponding author. The data are not publicly available due to privacy or ethical restrictions.
