# Supplementary material for: Combining Detailed Fetal Anatomy Scanning in the NT Window Versus Early Second Trimester Scanning at 14–16 Weeks: A Non‐Inferiority Study
Source: J Ultrasound Med. 2025 Aug 26;45(1):105–13. doi: 10.1002/jum.70036 (PMC12669442; doi:10.1002/jum.70036)
Supplement: Supplementary file 1 — Supplemental Table 1. A systematic approach to detailed assessment of the fetal anatomy at 11+0 to14+0 weeks should include the following. [file JUM-45-105-s001.docx]

**Supplemental Table 1:** A systematic approach to detailed assessment of the fetal anatomy at 11^+0^ to14^+0^ weeks should include the following.

| Anatomical region | Structures that can potentially be visualized in detailed anatomic survey |
| --- | --- |
| General | Conﬁrm singleton pregnancy  Overview of fetus, uterus and placenta |
| Head and brain | Calciﬁcation of cranium  Contour/shape of cranium (with no bony defects)  Two brain halves separated by interhemispheric falx  Choroid plexuses almost ﬁlling lateral ventricles in their posterior two-thirds (butterﬂy sign)  Thalami  Brainstem  Cerebral peduncles with aqueduct of Sylvius  Intracranial translucency (fourth ventricle)  Cisterna magna |
| Face and neck | Forehead  Bilateral orbits  Nasal bone  Maxilla  Retronasal triangle  Upper lip  Mandible  Nuchal translucency thickness  No jugular cysts in neck |
| Thorax | Shape of the thoracic wall  Lung ﬁelds  Diaphragmatic continuity |
| Heart | Heart activity present with regular heart rhythm  Establish situs  Position: intrathoracic heart position with cardiac axis to left (30–60◦)  Size: one-third of thoracic space  Four-chamber view with two distinct ventricles on grayscale and color Doppler in diastole  Left ventricular outﬂow tract view on grayscale or color Doppler  Three-vessel-and-trachea view on grayscale or color Doppler  Absence of tricuspid regurgitation/antegrade ductus venosus A-wave on pulsed-wave Doppler |
| Abdomen | Stomach: normal position in left upper abdomen  Bladder: normally ﬁlled in pelvis (longitudinal diameter < 7 mm)  Abdominal wall: intact with umbilical cord insertion  Two umbilical arteries bordering bladder  Kidneys: bilateral presence |
| Spine | Regular shape and continuity of spine |
| Extremities | Upper limbs with three segments and free movement  Lower limbs with three segments and free movement |
| Placenta | Size and texture normal, without cystic appearance  Location in relation to cervix and to previous uterine Cesarean section scar  Cord insertion into placenta |
| Amniotic ﬂuid and membranes | Amniotic ﬂuid volume  Amniotic membrane and chorion dissociated physiologically |

Reproduced with permission from Reference^1^

1. Bilardo CM, Chaoui R, Hyett JA, et al. ISUOG Practice Guidelines (updated): performance of 11-14-week ultrasound scan. Ultrasound Obstet Gynecol 2023;61:127-43.
